# Polysaccharide-Zein Composite Nanoparticles for Enhancing Cellular Uptake and Oral Bioavailability of Curcumin: Characterization, Anti-colorectal Cancer Effect, and Pharmacokinetics

**DOI:** 10.3389/fnut.2022.846282

**Published:** 2022-03-02

**Authors:** Lu Liu, Shufang Yang, Feng Chen, Ka-Wing Cheng

**Affiliations:** ^1^Institute for Food and Bioresource Engineering, College of Engineering, Peking University, Beijing, China; ^2^Shenzhen Key Laboratory of Marine Microbiome Engineering, Institute for Advanced Study, Shenzhen University, Shenzhen, China; ^3^Institute for Innovative Development of Food Industry, Shenzhen University, Shenzhen, China

**Keywords:** curcumin, polysaccharide-zein composite nanoparticles, cellular uptake, colorectal cancer, pharmacokinetics

## Abstract

Curcumin (CUR) has demonstrated promising potential as a therapeutic agent against colorectal cancer (CRC). However, its intrinsic shortcomings, including oxidative instability, sensitivity to gastrointestinal (GI) hydrolytic/enzymatic action, and susceptibility to biotransformation and systemic elimination, have greatly undermined its value for application in clinical settings. The development of carriers, in particular oral formulations, for its efficient delivery has remained an important direction in nutraceutical research. In the present work, CUR-encapsulated nanoparticles were fabricated with zein alone (Zein-CUR) and with zein and a polysaccharide (PS) [gum Arabic (GA), hyaluronic acid (HA) and pectin (PC), respectively] (PS-Zein-CUR). Their physicochemical and biological properties were evaluated in a series of *in vitro* and *in vivo* assays. Dynamic light scattering analysis showed an increase in the particle size of the nanoparticles from 129.0 nm (Zein-CUR) to 188.8–346.4 nm (PS-Zein-CUR). The three PS-Zein-CUR formulations had significantly higher (17–22%) CUR encapsulation efficiency (EE) than Zein-CUR. Among them, HA-Zein-CUR exhibited the highest EE and loading capacity. Zeta potential and FTIR spectra indicated the involvement of electrostatic and hydrophobic interactions and hydrogen bonds in the formation of the PS-Zein-CUR. In human CRC cell lines (HCT8, HCT29, and HCT116), the three PS-Zein-CUR and CUR all effectively inhibited cell viability and colony formation (HA-Zein-CUR > PC-Zein-CUR > GA-Zein-CUR/CUR). HA-Zein-CUR and PC-Zein-CUR also resulted in significantly higher cellular uptake of CUR than GA-Zein-CUR and CUR. Simulated GI-digestion assay demonstrated significantly improved controlled-release properties of these two formulations. Further pharmacokinetics and tissue distribution assays in a CRC subcutaneous xenograft model in nude mice corroborated the enhanced pharmacokinetic properties of intragastric administration of HA-Zein-CUR compared with that of free CUR (3 times higher C_*max*_ and 9.18 times higher plasma AUC). HA-Zein-CUR also led to enhanced delivery and accumulation of CUR in major organs/tissues, in particular CRC tumors and colon. These results together support that HA-Zein-CUR has promising potential as an oral agent for the control of CRC.

## Introduction

Curcumin (CUR), a natural polyphenol derived from turmeric (*Curcuma longa*), has been approved as a food additive in many countries. It has been reported to suppress the initiation, promotion, and/or progression of various types of cancers *via* multiple action mechanisms (1). In particular, its efficacy in colorectal cancer (CRC) control has been widely studied with promising results (2, 3). However, it has poor solubility and pH sensitivity, and is susceptible to biotransformation. These properties together with its sensitivity to the hydrolytic condition of the gastrointestinal (GI) tract have significantly compromised its oral bioavailability and limited its value for clinical applications (4). When administered orally to rats at a dose of 1 g/kg, 75% of CUR was excreted in the feces (5). In humans, oral administration of CUR at a dose of 2 g resulted in undetectable or extremely low serum levels (6). An array of carriers has been proposed to help overcome one or more of these shortcomings, and thus improve the efficacy of CUR in various disease models (7, 8).

Formulations for oral administration are among the most highly sought-after and have attracted a lot of research interest. In this regard, many studies have been centered on biocompatible, biodegradable, and highly safe natural molecules, such as proteins and polysaccharides (PS), for the fabrication of delivery systems for nutraceuticals (9, 10). As a major protein in corn, zein has been much appreciated for its low production cost besides being a renewable macromolecule. It is highly amphipathic and can easily self-assemble into nanoparticles by the antisolvent precipitation method, making it a favorable material for encapsulating hydrophobic bioactive compounds (11). Nonetheless, studies also showed that zein may form aggregates in the GI tract under near-isoelectric and high-ionic strength conditions, and may be partially digested by trypsin (12). Coating with PS, such as gum Arabic (GA), hyaluronic acid (HA), pectin (PC), chitosan, and alginate, has been proposed to be a promising approach to help overcome these limitations (13). These PS are capable of forming a core-shell structure with zein *via* crosslinking between the various functional groups on the macromolecules. Studies showed that PS-Zein had the advantages of convenient preparation, good dispersion and stability in aqueous solution, which make them ideal carriers to protect the encapsulated bioactive molecules from the chemical or biochemical insults of the external environment (14–16). However, most studies have focused on the fabrication and characterization of the physicochemical characteristics of PS-Zein. Information regarding the biological properties including bioactivity and pharmacokinetics of these promising nanocarriers remains limited.

**FIGURE 1:**
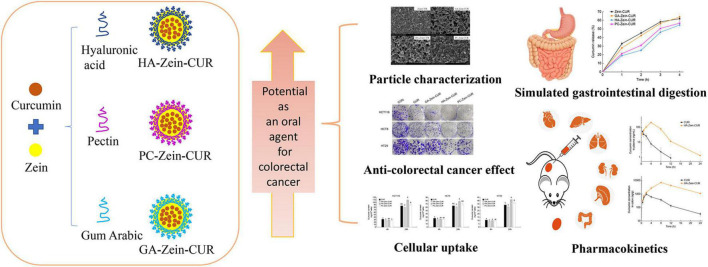
Experimental scheme. The evaluation of the potential of PS-Zein-CUR an oral agent for CRC therapy: physicochemical properties, anti-CRC effects, cellular uptake, and gastrointestinal simulated digestion *in vitro* and pharmacokinetics and tissue distribution *in vivo.*

The present study aimed to evaluate the potential of CUR-encapsulated PS-Zein nanoparticles (PS-Zein-CUR) as an oral agent for CRC therapy. Three common natural PS (GA, HA, and PC) which have wide applications in the food and pharmaceutical industries were chosen to fabricate the corresponding PS-Zein-CUR. Their physicochemical properties, anticancer effects, and cellular uptake in CRC cells were investigated. Furthermore, their simulated GI digestion (SGID) *in vitro* and pharmacokinetics and tissue distribution *in vivo* were also examined. A scheme of the experimental design is shown in Figure 1.

## Materials and Methods

### Materials

Curcumin was obtained from Tianjin Guangfu Fine Chemical Research Institute (Tianjin, China). HA (100–300 kDa, ≥99%) was purchased from Xi’an XABC Biotech Co., Ltd., (Xi’an, China). GA (51198), PC (P9135), and zein (Z3625) were purchased from Sigma-Aldrich (St. Louis, MO, United States). HCT116, HCT8, and HT29 cells were obtained from the National Infrastructure of Cell Line Resource (Beijing, China).

### Preparation of the Composite Nanoparticles

Zein-CUR and PS-Zein-CUR were prepared using an antisolvent coprecipitation method based on a previous study with some modifications (17). Briefly, zein (100 mg) and CUR (10 mg) were dissolved in 10 mL of aqueous ethanol (80%, v/v). The solution was slowly injected into 50 mL of distilled water or a PS solution (GA: 3 mg/mL; HA: 0.4 mg/mL; or PC: 0.6 mg/mL) with stirring at 600 rpm for 20 min. The mass ratios of PS to zein in the formulations were chosen based on a few recent reports (17–19). The ethanol was removed from the solution by rotary evaporation (45°C, −0.1 MPa). This was followed by centrifugation at 3,000 rpm for 10 min to remove the insoluble particles. The blank Zein/PS-Zein without CUR were also prepared with the same protocol.

### Characterization of Composite Nanoparticles

After appropriate dilution, the composite nanoparticles were analyzed in a dynamic light scattering (DLS) particle analyzer (NanoBrook 90Plus, Brookhaven, GA, United States) to determine their particle size, polydispersity index (PDI), and zeta potential. The turbidity of the nanoparticle suspensions was analyzed by measuring their optical density at 600 nm (OD_600_) in a spectrophotometer (NP80 Touch, Implen, Munich, Germany). Their micromorphology was captured with a field-emission scanning electron microscope (FE-SEM) (SU8020, HITACHI, Tokyo, Japan), and their Fourier transform infrared (FTIR) spectra were recorded with an FTIR spectrometer (IS10, Nicolet, Wisconsin, WI, United States) in the wavenumber range of 400–4,000 cm^–1^. Sixty-four scans at a resolution of 4cm^–1^ were performed for each sample. CUR in the nanoparticles was extracted with ethanol and quantified at 426 nm in a microplate reader (SpectraMaxTM i3X, Molecular Devices, Silicon Valley, CA, United States) using a standard curve. Encapsulation efficiency (EE) and loading capacity (LC) of the CUR-encapsulated nanoparticles were calculated using the following equations:

EE = encapsulated CUR (g)/total CUR (g) × 100%

LC = encapsulated CUR (g)/total mass of nanoparticles (g) × 100%

### Cell Viability Assay

Cytotoxicity of CUR, PS-Zein and PS-Zein-CUR against CRC cells was assessed by using a CCK-8 kit (CK-04, Dojindo, Kumamoto, Japan). Briefly, cells were seeded in 96-well plates at a density of 5 × 10^3^ cells per well. After 24 h of incubation at 37°C, the cells were treated with the above agents for 48 h, and then incubated with CCK-8 solution for 1 h. Absorbance was measured at 450 nm in a microplate reader. The IC_50_ values of the different treatments were determined using the logistic regression model in SPSS software.

### Colony-Formation Assay

A thousand cells were seeded into each well of a 6-well plate and allowed to attach. After 48-h treatment with drugs or vehicles, the cells were cultured in media without drugs for 2 weeks. The colonies formed were fixed with methanol, stained with 0.1% crystal violet, and counted to obtain an average sum.

### Cellular Uptake Analysis

Cells were seeded in 6-well plates at a density of 5 × 10^5^ cells per well. After incubation with drugs for 4 and 24 h, respectively, the cells were harvested and washed twice with PBS. CUR was extracted from the cells with aqueous acetonitrile (70%) assisted with ultrasonication. The extract was centrifuged at 14,000 rpm for 10 min (4°C), and the supernatant was filtered through a 0.22-μm membrane prior to UPLC-MS analysis.

### UPLC-MS Analysis of Curcumin

UPLC-MS analysis of CUR was conducted on a ACQUITY UPLC system coupled with a triple-quadrupole mass spectrometer (Waters, Milford, MA, United States) and equipped with a BEH reversed-phase C18 column (50 × 2.1 mm, 1.7 μm, Waters). The mobile phase consisted of acetonitrile (A) and water with 0.1% formic acid (B) at a flow rate of 0.3 mL/min. The injection volume was 1 μL. The gradient elution program was as follows: 0–6 min 40–100% A, 6–8 min, 100–40% A. MS/MS data acquisition was performed under positive electrospray ionization mode. Multiple Reaction Monitoring mode was employed to monitor CUR with precursor-to-product ion transition of *m/z* 369.28/145.13 and *m/z* 369.28/177.12. The MS parameters were set as follows: capillary voltage 3.0 Kv, desolvation gas flow 600 L/h, desolvation temperature 400°C, and cone voltage 20 V.

### Simulated Gastrointestinal Digestion Assay

Simulated gastrointestinal digestion (SGID) assay was carried out by the method of Brodkorb et al. (20). Briefly, samples were diluted with an equal volume of simulated gastric fluid (SGF) and incubated in a shaking water bath (100 rpm, 37°C) for 2 h. The gastric chyme was then diluted with an equal volume of simulated intestinal fluid (SIF) and incubated in a shaking water bath (100 rpm, 37°C) for a further 2 h. Samples were collected at 1, 2, 3, and 4 h during SGID and then centrifuged with Amicon Ultra centrifugal filters (MWCO 3 kDa). CUR was quantified by UPLC-MS.

### Pharmacokinetics and Tissue Distribution Study

All animal experiments were approved by the Animal Ethical and Welfare Committee of Shenzhen University and carried out in accordance with the Regulations for the Administration of Affairs Concerning Experimental Animals of China. BALB/c nude mice (6-week-old, female) were purchased from Guangdong Medical Laboratory Animal Center (Foshan, China). The mice were kept under pathogen-free conditions and allowed to acclimatize for a week before experimentation. HCT116 cells (suspended in serum-free medium containing 50% Matrigel) were injected subcutaneously into the right flanks of the BALB/c nude mice at a density of 1 × 10^6^ cells per mouse. Five days after implantation when the HCT116-derived xenografts were established, the mice were randomly divided into two groups (21 mice per group) for the following intragastric treatments, respectively: CUR (6 mg/kg, dissolved in 0.5% sodium carboxymethylcellulose) and HA-Zein-CUR (equivalent to 6 mg/kg CUR). Following administration, three mice were sacrificed at each of the designated time points (0.5, 1, 2, 4, 8, 12, and 24 h), and their blood, tumors, and organs (heart, lung, liver, colon, kidney, and spleen) were collected. The blood samples were centrifuged at 4,000 rpm at 4°C for 10 min to obtain plasma. Two hundred microliters of each plasma sample were used for extraction. The tumors and organs were washed and homogenized with precooled PBS in a TissueLyser (85300, Qiagen, Dusseldorf, Germany). Acetonitrile (3x, v/v) was used to extract CUR from plasma and tissue homogenates assisted with ultrasonication. After centrifugation at 14,000 rpm for 10 min (4°C), the supernatant was filtered through a 0.22-μm membrane and then analyzed by UPLC-MS.

### Statistical Analysis

The data of the experiments are presented as mean ± SD. Significant differences among groups were identified by one-way ANOVA and the Duncan test. SPSS software was used for the analyses. *P* < 0.05 was considered to be statistically significant.

## Results and Discussion

### Dynamic Light Scattering, Encapsulation Efficiency, and Loading Capacity Analysis of Curcumin-Loaded Composite Nanoparticles

**FIGURE 2:**
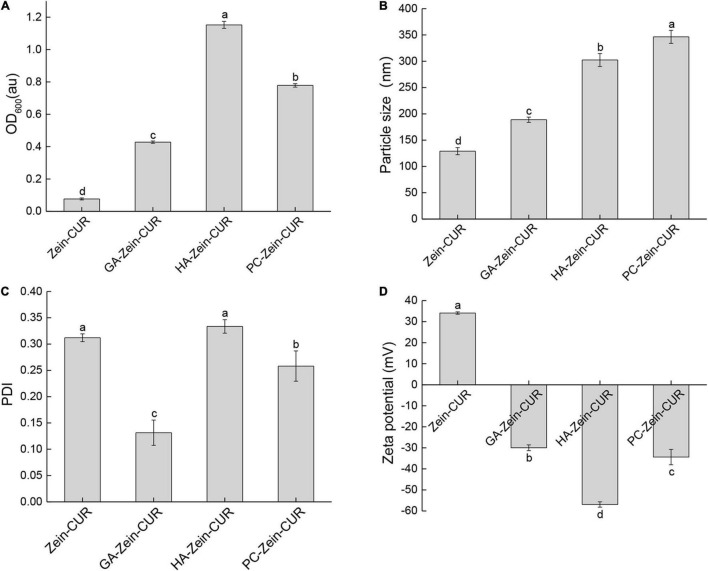
Analysis of zein-curcumin (CUR) and polysaccharide (PS)-Zein-CUR suspensions. **(A)** Turbidity (OD_600_), **(B)** particle size, **(C)** polydispersity index (PDI), and **(D)** zeta potential. Values are mean ± SD, *n* = 3. Bars marked with different letters indicate significant difference at *P* < 0.05.

The nanoparticle samples were subjected to DLS analysis of particle size, PDI, and zeta potential. As shown in Figure 2A, the incorporation of PS (GA, HA, or PC) significantly increased the turbidity (OD_600_) of the Zein-CUR formulation. The adsorption of the anionic PS onto the surface of the cationic zein molecules also led to an increase in the particle size of the nanoparticles from 129.0 nm (Zein-CUR) to 188.8–346.4 nm (PS-Zein-CUR) (Figure 2B). HA-Zein-CUR and PC-Zein-CUR had significantly larger particle sizes than GA-Zein-CUR, which likely explained the higher turbidity of the former solutions than the latter (21). The particle size of PS-Zein-CUR was dependent on the inherent attribute of PS, such as electric charge (17). The PDI values of GA-Zein-CUR, HA-Zein-CUR, and PC-Zein-CUR were 0.13, 0.33, and 0.25, respectively, suggesting a narrower particle size distribution of GA-Zein-CUR than the other two formulations (Figure 2C). This is consistent with the results of a recent study that the presence of GA altered the polydispersity of Zein nanoparticles (22). It was also observed that the addition of PS greatly altered the surface charge density of the nanoparticles as evidenced by the reversal of the zeta potential from positive on Zein-CUR (34.1 mV) to negative on GA-Zein-CUR, HA-Zein-CUR, and PC-Zein-CUR (−30.0, −56.9 and −34.4 mV, respectively) (Figure 2D). The low negative zeta potentials (< −30 mV) also indicate that PS-Zein-CUR had good stability, especially HA-Zein-CUR, which was likely due to the capability of HA to introduce stronger electrostatic repulsive forces to the nanoparticles than GA and PC (23).

The EE and LC of the nanoparticles are presented in Table 1. The incorporation of the PS (GA, HA, or PC) significantly increased the EE of the Zein-CUR by 17 – 22%. A similar phenomenon was also reported for resveratrol-encapsulated Zein nanoparticles, whose EE was improved by chitosan coating (24). Meanwhile, LC of the formulations did not seem to have any particular trend. Only HA was able to moderately (9%) improve LC of the nanoparticles, while PC did not have any significant effect, and GA led to a significant reduction of LC by ∼50%. Taken together the EE and LC data, HA-Zein-CUR was thus considered to be the most effective carrier for CUR among the three formulations.

**TABLE 1:** Encapsulation efficiency (EE) and loading capacity (LC) of CUR-loaded composite nanoparticles.

	EE (%)	LC (%)
Zein-CUR	75.59 ± 2.57^c^	6.87 ± 0.23^B^
GA-Zein-CUR	92.94 ± 3.15^b^	3.57 ± 0.12^C^
HA-Zein-CUR	97.24 ± 2.23^a^	7.48 ± 0.17^A^
PC-Zein-CUR	95.05 ± 1.74^ab^	6.79 ± 0.12^B^

*Values are mean ± SD, n = 3. Different letters in the same column indicate significant difference at P < 0.05.*

### Spectroscopic Analysis of PS-Zein-CUR

**FIGURE 3:**
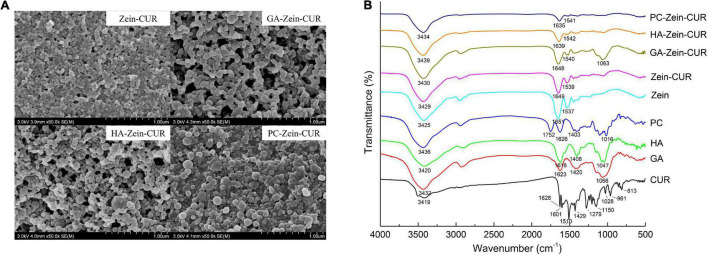
Spectroscopic analysis of composite nanoparticles. **(A)** FE-SEM images (50,000× magnification) of Zein-CUR and PS-Zein-CUR. **(B)** FTIR spectra of CUR, PS, Zein, Zein-CUR, and PS-Zein-CUR.

Representative FE-SEM images of Zein-CUR and PS-Zein-CUR are shown in Figure 3A. The Zein-CUR appeared to be closely packed spheres, and the PS-Zein-CUR were in more loosely arranged bigger spheres. The GA-Zein-CUR seemed to have more uniform and smaller particle sizes than the HA-Zein-CUR and PC-Zein-CUR, which was consistent with the DLS data showed in Figures 2B,C. The generally bigger size and looser arrangement of the PS-Zein-CUR than the Zein-CUR was likely due to the intermolecular interactions of the PS molecules, in particularly hydrogen bonding among -COOH, -NHCOCH_3_, and -OH groups (25, 26).

FTIR spectroscopy was subsequently performed to provide some theoretical grounds for the purported intermolecular interactions in the nanoparticle formulations. The FTIR spectra of CUR, PS, Zein, Zein-CUR, and PS-Zein-CUR are displayed in Figure 3B. The Zein-CUR showed characteristic peaks at 3,429, 1,649, and 1,539 cm^–1^, which could be assigned to the vibration of hydroxyl, amide I, and amide II groups, respectively (27). It was noticed that the Zein and Zein-CUR exhibited almost identical IR spectra. This was likely due to the masking effect of the characteristic peaks of CUR (1,628, 1,601, and 1,510 cm^–1^) by the amide I and amide II peaks of Zein. The identical IR spectra together with the almost complete disappearance of CUR’s characteristic peaks at the regions of 813–1,429 cm^–1^ also suggests that CUR was well encapsulated and did not appreciably change the primary structure of Zein (28). The blue shift of the peaks corresponding to hydroxyl stretching vibration of the PS-Zein-CUR (3,430–3,439 cm^–1^) relative to the Zein-CUR (3,429 cm^–1^) may indicate the formation of hydrogen bonds between the hydroxyl groups of the PS and the amide groups of zein that resulted in lower bond energy than the original hydrogen bonds in the Zein-CUR formulation (29). Chen et al. also suggested that hydrogen bonding was a main driving force in the formation of zein-chitosan-quercetagetin nanoparticles (30). Meanwhile, the red shift of the amide I (1,635–1,648 cm^–1^) and blue shift of the amide II peak (1,540–1,542 cm^–1^) in the PS-Zein-CUR relative to those in the Zein-CUR (1,649 and 1,539 cm^–1^, respectively) may arise as a result of hydrogen bond formation as well as hydrophobic interactions between zein and the PS molecules (31). Zein is known to have a high content of hydrophobic amino acids (32). The presence of GA, HA, or PC would change the hydrophilicity/hydrophobicity property of the surrounding environment of the zein polypeptides, and thus perturb their hydrophobic interaction (31). In addition, the disappearance of the asymmetric and symmetric COO- peaks of anionic PS (1,618–1,626 cm^–1^ and 1,403–1,420 cm^–1^) in the PS-Zein-CUR also supports the occurrence of crosslinking between the PS and zein molecules (33). The C-O-C stretch peaks of the PS (1,016–1,066 cm^–1^) was retained in the GA-Zein-CUR, but nearly disappeared in the HA-Zein-CUR and PC-Zein-CUR. This might be attributed to the lower mass ratio of HA/PC to zein that led to a stronger shielding effect on the PS C-O-C groups.

### Effect of Curcumin-Loaded Composite Nanoparticles on Human Colorectal Cancer Cells

**FIGURE 4:**
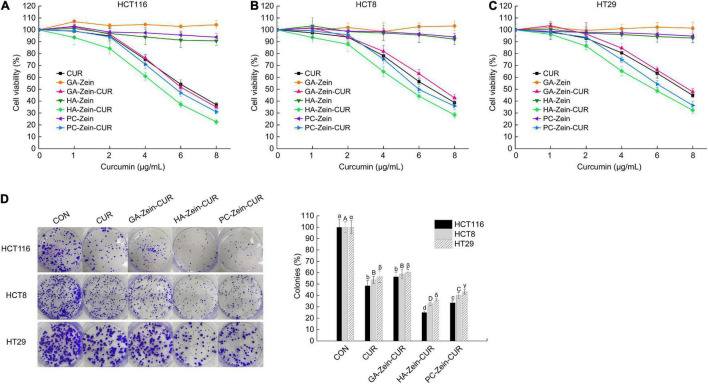
Inhibitory effect of PS-Zein-CUR on CRC (HCT116, HCT8, and HT29) cell growth. Cells were treated with the indicated doses of CUR, PS-Zein, and PS-Zein-CUR for 48 h, respectively. **(A–C)** Cell viability measured by using a CCK-8 cell counting kit. **(D)** Left: images of colony formation assay; Right: quantitative analysis of colony formation expressed as percentage relative to vehicle control. Values are mean ± SD, *n* = 3. Bars marked with different letters indicate significant difference at *P* < 0.05.

The inhibitory effects of CUR, PS-Zein, and PS-Zein-CUR on the viability of human CRC cells were evaluated in HCT116, HCT8, and HT29 cell lines (Figures 4A–C). As expected, the blank PS-Zein did not show any significant inhibitory effect on CRC cell viability over the concentration range assayed. CUR and PS-Zein-CUR, on the other hand, all showed strong and dose-dependent growth inhibitory effects in the three cell lines. However, they did not seem to have any selective effect toward any of the three cell lines. As shown in Table 2, the order of their IC_50_ values in the three cell lines was CUR/GA-Zein-CUR > PC-Zein-CUR > HA-Zein-CUR, indicating significantly stronger cytotoxicity of HA-Zein-CUR than the other two formulations. Based on the cell viability assay data, 4 μg/mL of CUR and PS-Zein-CUR (<IC_50_) were selected to subsequently assess their inhibitory effect on colony formation of the CRC cells. The order of their inhibitory effect was HA-Zein-CUR > PC-Zein-CUR > CUR/GA-Zein-CUR (Figure 4D), which was consistent with the result of the cell viability assay. These data suggest that the encapsulation of CUR in PC-Zein and HA-Zein, but not GA-Zein, led to enhanced inhibitory effect on CRC cells. Considering the negligible cytotoxicity of the blank nanocarriers themselves toward the CRC cells, it was hypothesized that the differential inhibitory effects of CUR and the PS-Zein-CUR may be associated with their different cellular uptake efficiencies.

**TABLE 2:** IC_50_ values of CUR and PS-Zein-CUR in HCT116, HCT8, and HT29 cells.

IC_50_ (μ_g/mL)	HCT116	HCT8	HT29
CUR	6.40 ± 0.68^a^	6.73 ± 0.82^A^	7.55 ± 1.12^α^
GA-Zein-CUR	6.24 ± 0.59^a^	7.32 + 0.98^A^	7.79 ± 1.07^α^
HA-Zein-CUR	4.61 ± 0.51^b^	5.24 ± 0.63^B^	5.58 ± 0.70^β^
PC-Zein-CUR	5.76 ± 0.54^a^	6.19 ± 0.60^AB^	6.40 ± 0.73^α^_^β^

*Values are mean ± SD, n = 3. Different letters in the same column indicate significant difference at P < 0.05.*

**FIGURE 5:**
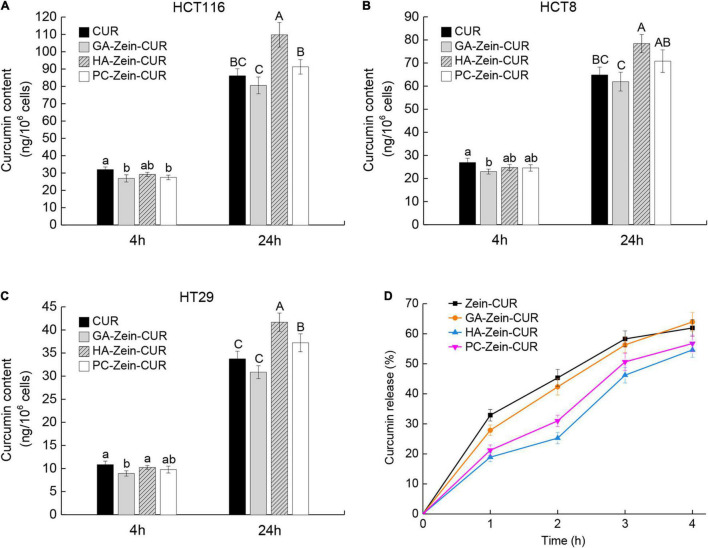
Cellular uptake and simulated gastrointestinal digestion (SDIG) of PS-Zein-CUR. **(A–C)** CUR contents in CRC cells incubated, respectively with CUR and PS-Zein-CUR for 4 and 24 h. Values are mean ± SD, *n* = 3. Bars marked with different letters indicate significant difference at *P* < 0.05. **(D)** CUR release from Zein-CUR and PS-Zein-CUR during SDIG. Values are mean ± SD, *n* = 3.

Results of the cellular uptake of CUR and PS-Zein-CUR are presented in Figures 5A–C. Compared with free CUR, PS-Zein-CUR did not promote (GA even decreased) the uptake or accumulation of CUR in the CRC cells after incubation for 4 h. However, after incubation for 24 h, HA-Zein-CUR significantly increased CUR content in HCT116, HCT8, and HT29 cells, and PC-Zein-CUR significantly increased it in HT29 cells, while GA-Zein-CUR did not result in any increase of CUR content in the three CRC cell lines. The different cellular uptake of CUR could be attributed to the time-dependent controlled release and internalization mechanisms of the PS-Zein-CUR, which depend on the inherent properties of the PS. The encapsulation of CUR in PS-Zein-CUR likely slowed down CUR degradation in the culture medium (34). In addition, different from CUR, PS-Zein-CUR might enter the cells through endocytosis, which resulted in more efficient cellular uptake under the experimental conditions (35). In particular, specific ligand-receptor interactions between nanoparticles and cancer cells have been considered a promising strategy to enhance the cellular uptake of pharmaceuticals (36). HA has been reported to be capable of specifically interacting with certain proteins overexpressed in cancer cells, such as CD44, Rhamm, and TSG6, thus facilitating the targeting of HA-fabricated nanoparticles to cancer cells (37). Parashar et al. found that HA decorated poly caprolactone nanoparticles could increase the delivery efficiency of naringin to lung cancer cells by actively targeting the overexpressed CD44 (38).

### Simulated GI Digestion of Curcumin-Loaded Nanoparticles

Prior to further evaluation of the pharmacokinetics of the CUR-loaded nanoparticles, their stability under simulated GI condition was assessed (Figure 5D). A high release rate (25.88%) of CUR from Zein-CUR was observed during the 1st h of the SGID. The release gradually slowed down toward later time points, reaching 61.93% at 4 h. GA-Zein-CUR exhibited similar release characteristics to Zein-CUR. In contrast, HA-Zein-CUR and PC-Zein-CUR had significantly lower release rates than Zein-CUR during the SGID. The much better CUR-retention capability of HA-Zein-CUR and PC-Zein-CUR (44.26 and 26.75% higher than Zein-CUR, respectively) could be attributed to the PS coating that protected zein from the hydrolytic action of pepsin (12). The difference in their release rates tended to get smaller in the intestinal digestion phase. This was mainly due to the accelerated release of CUR from HA-Zein-CUR and PC-Zein-CUR during 2–3 h upon transmit from the simulated gastric to intestinal condition, while the release rates of the other two formulations remained steady. These results indicate that HA-Zein-CUR and PC-Zein-CUR may help reduce the loss of CUR under gastric condition, and allow its controlled release during intestinal digestion.

### Pharmacokinetics and Tissue Distribution of Free Curcumin and HA-Zein-CUR in Mice

**FIGURE 6:**
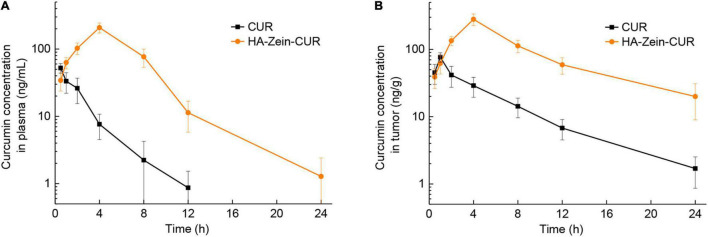
Pharmacokinetics analysis of free CUR and HA-Zein-CUR in mice. **(A)** Plasma and **(B)** tumor concentration profiles of CUR in mice following intragastric administration of free CUR and HA-Zein-CUR, respectively. Values are mean ± SD, *n* = 3.

Among the three PS-Zein-CUR, HA-Zein-CUR exhibited the best inhibitory effect and cellular uptake in the CRC cell lines. It also demonstrated better controlled release of CUR than GA- and PC-Zein-CUR during the SGID. These results support its good potential as an efficient oral delivery system for CUR in CRC treatment. Subsequently, a CRC (HCT116) xenograft model in nude mice was employed to further evaluate its pharmacokinetics (Figure 6A and Table 3). Following intragastric administration of HA-Zein-CUR, the plasma concentration of CUR increased gradually and reached 208.37 μg/L (C_*max*_) at 4 h, which was threefold higher than that achieved with the administration of free CUR (76.73 ± 12.27 ng/g). T_*max*_ of free CUR was at 0.5 h. These data further supported the successful sustained-release behavior of HA-Zein-CUR, which was also consistent with the SGID data *in vitro*. Moreover, HA-Zein-CUR had a 1.70 times longer t_1/2_ and 2.68 times longer mean residence time (MRT) than those of free CUR, indicating significantly slower systemic elimination of the former. The superior pharmacokinetic profile of CUR accomplished with HA-Zein-CUR when compared to that accomplished with free CUR was also reflected in the much higher (9.18 times) area under curve (AUC_0–*t*_) of the former than the latter. In addition, according to the equation of a previous study (39), the relative bioavailability (RB) of HA-Zein-CUR compared to free CUR was calculated to be 10.18. This was higher than those of formulations such as MicroActive CUR (RB = 9.7) and Micronized CUR (RB = 9), which were reported to have significantly improved bioavailability over free CUR (40, 41). These data together therefore suggested that the formulation was able to substantially increase the oral bioavailability of CUR. Over the last decade, many formulations, such as liposomes, phytosomes, piperine complex, cyclodextrin inclusions and colloidal nanoparticles, have been developed to improve the bioavailability of CUR mainly *via* increasing its solubility and gastrointestinal stability (39). Compared with most existing formulations, our formulation was prepared using a simple antisolvent coprecipitation method and consisted of only natural macromolecules zein and HA, and thus had the advantage of easy preparation and high safety as an oral agent.

**TABLE 3:** Pharmacokinetic parameters of free CUR and HA-Zein-CUR after intragastric administration in mice.

Parameter	CUR	HA-Zein-CUR
C_max_ (μg/L)	52.27 ± 8.66	208.37 ± 28.16
T_max_ (h)	0.50	4.00
t_1/2_ (h)	2.07 ± 0.61	3.57 ± 0.94
MRT (h)	2.68 ± 0.43	5.92 ± 0.52
AUC_0–t_ (μg/L⋅h)	123.90 ± 8.32	1261.23 ± 75.24
AUC_0–8_ (μg/L⋅h)	126.50 ± 8.38	1281.35 ± 76.13
RB	1	10.18

*Values are mean ± SD, n = 3. t of AUC_0–t_ is 24 h. RB, relative bioavailability.*

Having established the favorable pharmacokinetic behavior of the HA-Zein-CUR, we proceeded to examine its tissue distribution in the xenograft model. Compared to free CUR, intragastric administration of HA-Zein-CUR resulted in a significantly higher C_*max*_ of CUR in the tumors (280.33 ± 54.86 vs. 76.73 ± 12.27 ng/g) and a longer T_*max*_ (4 h vs. 1 h) (Figure 6B). A previous report also demonstrated that HA-Zein nanoparticles loaded with hydrophobic agents were capable of accumulating in colon carcinoma CT26 tumors although the test agent was given intravenously to the mice (42). Our data together with the literature therefore strongly support the favorable pharmacokinetic properties of the HA-Zein-CUR.

**FIGURE 7:**
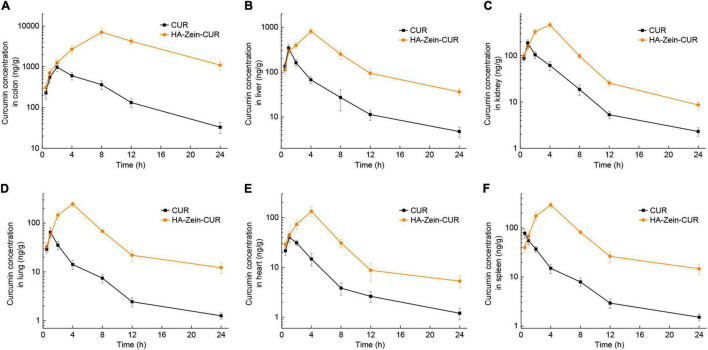
Organ distribution of CUR in mice. Concentration profile of CUR in panel **(A)** colon, **(B)** liver, **(C)** kidney, **(D)** lung, **(E)** heart, and **(F)** spleen following intragastric administration of free CUR and HA-Zein-CUR, respectively. Values are mean ± SD, *n* = 3.

Measurement of the CUR levels in major organs after intragastric administration of HA-Zein-CUR or free CUR showed distinct delivery/accumulation of CUR between these two agents (Figure 7). For HA-Zein-CUR, C_*max*_ appeared at 4 h in all the organs assayed except colon. For free CUR, C_*max*_ occurred at more varied and earlier time points (0.5–2 h) in the different organs. It was also noted that the C_*max*_ values of both free CUR and HA-Zein-CUR in the colon were significantly higher than in other organs. Starting from the 2-h till the 24-h time point, HA-Zein-CUR exhibited much better delivery efficiency than free CUR. Remarkably, the C_*max*_ (7070.72 ± 1509.59 ng/g) in the colon achieved with HA-Zein-CUR was 6.19-fold higher than that achieved with free CUR. Even after 24 h, CUR concentration in the colon following administration of HA-Zein-CUR remained at 1101.44 ± 291.84 ng/g, which was 32.44-fold higher than that in the free CUR group. These data were also consistent with the time-course distribution analysis in the xenografts of the mice.

Curcumin has been reported to inhibit the proliferation of CRC cells *via* regulating multiple signaling pathways (43). Delivery of effective dosages to the tumor cells or target tissue *in vivo*, however, has remained a major challenge for the realization of the purported anticancer efficacy, and thus its application in clinical settings. The results from the pharmacokinetics and tissue distribution assay have provided strong evidence that the HA-Zein-CUR could deliver and sustain high levels of CUR in the subcutaneous CRC tumors. Its preferential distribution to the colon over other tissues/organs also indicates a promising therapeutic potential against orthotopic CRC tumors.

## Conclusion

In conclusion, coating of Zein-CUR with GA, HA, and PC had significant and distinct effects on the physicochemical properties of the nanoparticles, including particle size, colloidal stability, EE, and LC. The controlled-release property and enhanced cellular uptake of the PS-Zein-CUR were translated into their significantly stronger activity against the growth of multiple CRC cell lines. Among them, HA-Zein-CUR was the most promising and its favorable pharmacokinetic behavior and distribution profile were further corroborated *in vivo*, in particular the multi-fold enhancement in its delivery to the tumors as well as the colon. The results from the SGID, cellular uptake, and pharmacokinetics and tissue distribution assays in the present study highlight that the HA-Zein-CUR represents a promising oral delivery system for further evaluation of the therapeutic value of CUR in diverse CRC models including subcutaneous xenograft and orthotopic models.

## Data Availability Statement

The raw data supporting the conclusions of this article will be made available by the authors, without undue reservation.

## Ethics Statement

The animal study was reviewed and approved by the Animal Ethical and Welfare Committee of Shenzhen University.

## Author Contributions

LL and K-WC conceived and designed the experiments. LL and SY performed the experiments. LL, SY, and K-WC revised the manuscript. K-WC and FC acquired the funding and supervised the study. All authors contributed to the article and approved the submitted version.

## Conflict of Interest

The authors declare that the research was conducted in the absence of any commercial or financial relationships that could be construed as a potential conflict of interest. The reviewer RF declared a shared affiliation with one of the author LL at the time of the review.

## Publisher’s Note

All claims expressed in this article are solely those of the authors and do not necessarily represent those of their affiliated organizations, or those of the publisher, the editors and the reviewers. Any product that may be evaluated in this article, or claim that may be made by its manufacturer, is not guaranteed or endorsed by the publisher.
